# Supplementary material for: Mapping multi-modal dynamic network activity during naturalistic music listening
Source: Imaging Neurosci (Camb). 2025 Jan 2;3:imag_a_00413. doi: 10.1162/imag_a_00413 (PMC12319744; doi:10.1162/imag_a_00413)
Supplement: Supplementary Material [file imag_a_00413-supp.pdf]

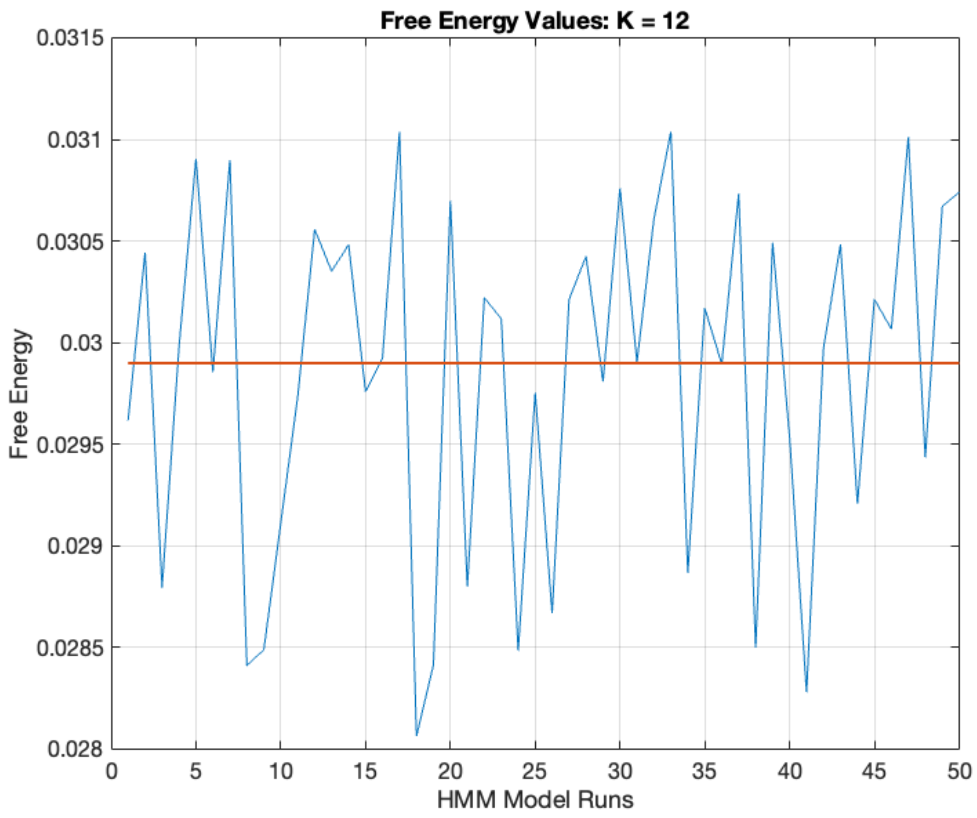

Supplementary Figure 1: Free energy values for 50 estimations of K = 12. The mean is 0.03 and is plotted as a horizontal bar. The standard deviation is 0.0008

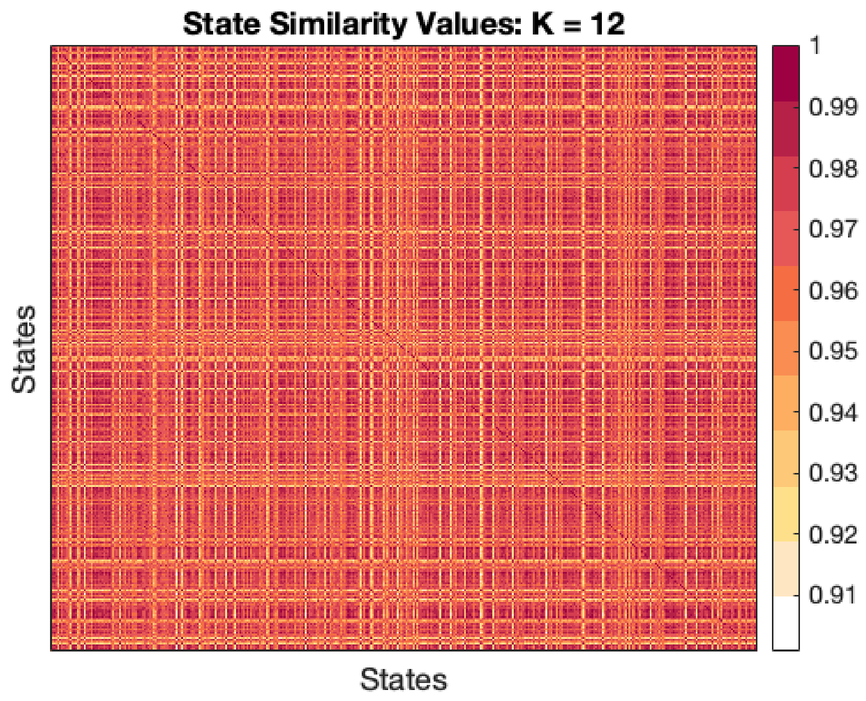

Supplementary Figure 2: Similarity matrix from STATIS analysis showing the state-wise similarity for each of 50 estimations at K = 12
